# Association of RAP Compensatory Reserve Index with Continuous Multimodal Monitoring Cerebral Physiology, Neuroimaging, and Patient Outcome in Adult Acute Traumatic Neural Injury: A Scoping Review

**DOI:** 10.1089/neur.2024.0058

**Published:** 2024-09-13

**Authors:** Abrar Islam, Izabella Marquez, Logan Froese, Nuray Vakitbilir, Alwyn Gomez, Kevin Y. Stein, Tobias Bergmann, Amanjyot Singh Sainbhi, Frederick A. Zeiler

**Affiliations:** ^1^ Biomedical Engineering, Faculty of Engineering, University of Manitoba, Winnipeg, Canada.; ^2^ Undergraduate Engineering, Biosystems Engineering, Faculty of Engineering, University of Manitoba, Winnipeg, Canada.; ^3^ Department of Human Anatomy and Cell Science, Rady Faculty of Health Sciences, University of Manitoba, Winnipeg, Canada.; ^4^ Undergraduate Medicine, Rady Faculty of Health Sciences, University of Manitoba, Winnipeg, Canada.; ^5^ Centre on Aging, University of Manitoba, Winnipeg, Canada.; ^6^ Pan Am Clinic Foundation, Winnipeg, Canada.; ^7^ Department of Clinical Neuroscience, Karolinska Institutet, Stockholm, Sweden.; ^8^ Section of Neurosurgery, Department of Surgery, Rady Faculty of Health Sciences, University of Manitoba, Winnipeg, Canada.

**Keywords:** acute traumatic brain injury, RAP, cerebral compliance, cerebral compensatory reserve, multimodal cerebral physiology, neuroimaging, patient outcomes

## Abstract

Acute traumatic neural injury, known as traumatic brain injury (TBI), stands as a significant contributor to global mortality and disability. Ideally, continuously monitoring cerebral compliance/cerebral compensatory reserve would enable timely interventions and avert further substantial deterioration in TBI cases. RAP, defined as the moving Pearson’s correlation between intracranial pressure (ICP) pulse amplitude waveform and ICP, has been proposed as a continuously updating index in this context. However, the literature remains scattered and difficult to navigate. Thus, the goal of this scoping review was to comprehensively characterize the literature regarding RAP and its association with (1) other multimodal cerebral physiological monitoring, (2) neuroimaging features, and (3) long-term patient outcomes. We subsequently conducted a systematic scoping review of the human literature to highlight the association of RAP with continuous multimodal monitoring of cerebral physiology, neuroimaging, and patient outcomes in the context of adult TBI patients. Our review encompassed 21 studies focusing on these topics. The primary findings involve meticulous analysis of studies, categorizing findings into three states of RAP to clearly understand its relation to cerebral physiology and clinical outcomes. State 1 signifies a healthy condition with a small positive value near zero (RAP <0.5). Conversely, state 2, a predominant characterization of TBI patients, indicates compromised compensatory reserve, featuring a large positive RAP value (RAP > 0.4). State 3 emerges in worsened conditions, showcasing further compromised compensatory reserve, exhausted cerebrovascular reactivity, and disturbed cerebral autoregulation. A substantial number of patients with fatal outcomes was found in state 3, marked by a notable occurrence of decreasing and, in some instances, negative RAP. The significance of this review lies in establishing a platform for future research directions to enhance the precision and clinical implications of RAP in TBI care, ultimately aiming to prevent the transition from state 2 to state 3 and mitigate fatal outcomes.

## Introduction

In the realm of neurocritical care, the continuous monitoring of cerebral physiological parameters plays a pivotal role since it can contribute extensively to the interpretation and management in various neurological conditions, as is the case in acute biomechanical neural injury, also termed traumatic brain injury (TBI).^1–3^ Cerebral compliance (CC)/cerebral compensatory reserve (CCR) is one such metric of significance, which offers insights into the brain’s capability of accommodating change in volume without any significant change in pressure.^4,5^ Continuous monitoring of CC**/**CCR potentially allows for the identification of abnormalities of cerebral physiological parameters, particularly that of intracranial pressure (ICP), at an early stage, facilitating timely interventions and preventing more significant deterioration.^1,6^ Among various techniques developed over the years for continuous CC/CCR, the RAP metric (correlation (R) between pulse amplitude of ICP (A) and ICP (P)) is one of the most researched metrics, offering vast opportunities for analysis and observation of its characteristics, patterns, and association with different parameters, thereby improving its precision to be applied in the clinical area of TBI care.

RAP is defined as the moving Pearson’s correlation of ICP pulse amplitude waveform (AMP) and ICP and ranges from −1 to +1.^4,5,7^ A good CC/CCR is indicated by a stable ICP, i.e., a lack of correlation between AMP and ICP. A stable small positive value of RAP represents good CC/CCR. With further increments in volume, ICP rapidly increases, showing a strong correlation between AMP and ICP. This represents a poor CC/CCR state, and RAP becomes close to +1.^5,8,9^ If ICP further elevates above 50 mmHg, there is a slight decrease in RAP values, as AMP and ICP show a negative correlation. This scenario suggests a compromised cerebral autoregulatory capacity, theorized vascular collapse (i.e., reaching critical closing pressure values), marking the final deterioration of cerebral blood flow continuity.^5,8,9^

However, existing literature on the RAP index in acute TBI cases remains scattered and difficult to navigate. In the case of moderate/severe TBI, insufficient research has been carried out to enable its clinical application. As a result, (1) the knowledge regarding the temporal statistical structure of RAP and (2) its association with other cerebral physiology in TBI cases is limited. Further to this, the link between RAP and both neuroimaging changes and long-term patient outcomes in TBI remains underreported. All of the above limit the ability for the RAP metric to be adapted into future prospective clinical studies/trials. As such, this systematic scoping review focuses on establishing such a foundation by thoroughly analyzing the current literature in this field.

This systematically conducted scoping review aims to comprehensively characterize the literature in three main areas. First is to comprehensively synthesize existing literature on the association of RAP with continuous multimodal monitoring (MMM) cerebral physiological parameters. This exploration will unravel the importance of the relationship between RAP and key variables as an indication of cerebral homeostasis. Second, the review addresses the association of RAP with neuroimaging findings, such as findings from computed tomography (CT) or magnetic resonance imaging (MRI), which is essential for bridging the gap between continuous monitoring data and structural brain changes, providing a more comprehensive understanding of the characteristics of RAP. Third, this review analyzes the association between RAP and patient outcome, providing insights into the potential prognostic implications of this metric. Understanding how RAP is related to outcome can be highly suggestive for tailoring therapeutic and recovery strategies, thereby aiding in preventing the exacerbation of the condition. By incorporating and critically evaluating the existing literature, this systematic scoping review aims to create a platform and guide future research directions to fill these gaps by enhancing the precision and illustrating valuable insights into the practicality of RAP as a bedside monitoring measurement in TBI care.

## Methods

This systematic review adhered to the guidance provided by the Cochrane Handbook for Systematic Reviews. The reporting of the review followed the recommendations outlined in the Preferred Reporting Items for Systematic Reviews and Meta-Analysis (PRISMA) guideline,^10^ including the PRISMA Extension for Scoping Review.^11^ The methodology and search resembled those utilized in previous systematic reviews conducted by the research team.^12,13^ The collaborative efforts of the primary authors (A.I. and I.M.) and senior author (F.A.Z.) formulated the review objectives and developed the search strategy. The PRISMA checklist can be found in Supplementary Appendix SA1.

### Search questions, population, and inclusion/exclusion criteria

The question addressed in this systematic review is as follows: What is the association between RAP and multimodal cerebral physiology, neuroimaging changes (CT, MRI), and patient outcome in adult TBI?

This systematic review encompasses all adult (age 18 and older) human studies that involve the observation of the association of RAP with continuous MMM cerebral physiological parameters, outcomes, and neuroimaging. There were no other limitations imposed based on sample size, patient characteristics, or data sampling method. There were studies where the association with RAP was not the primary focus but rather a factor contributing to the main objective, which were also considered and incorporated in this review.^6,7,9,14^

The exclusion criteria posed in this study were as follows: non-English language studies, animal studies, and theoretical studies, studies that are not exploring any association, studies not using RAP metric as the continuous CC/CCR measurement, nonoriginal studies, and abstract-only studies. Nonoriginal and abstract-only studies were excluded to prioritize significant and original research contributions in our analysis.

Acknowledging the crucial importance of RAP as a metric for assessing CC/CCR, this scoping review primarily aimed to investigate the patterns and features of RAP in diverse conditions. Consequently, studies that did not incorporate RAP as a metric or explore any associations with RAP were excluded. Animal studies were excluded because we aimed to comprehensively review the continuous CC/CCR measurement techniques directly applied to human subjects.

### Search strategy

Searches were conducted in major databases, including PubMed, Embase, Scopus, BIOSIS, and Cochrane Library, which covered records dating from the inception of each database up to mid-Dec 2023. Customized search strategies were developed for each database, detailed in Supplementary Appendix SA2. Furthermore, a thorough examination of the reference lists of the ultimately chosen studies was undertaken to ensure comprehensive coverage and prevent any oversight of relevant studies.

### Study selection

The study selection process involved a two-step review conducted by two reviewers, A.I. and I.M., for articles obtained from individual search strategies in each database. In the initial step, reviewers independently assessed all retrieved articles without sharing their progress. During this phase, decisions regarding inclusion or exclusion were based on the content of the title and abstract. The second step of study selection involved a thorough evaluation of full texts. We excluded studies that diverged from our main focus, which is to identify the correlation of RAP with continuous MMM cerebral physiological parameters, neuroimaging, and patient outcomes. Similar to the first step, this phase was conducted independently, and any disagreements between the two reviewers were resolved by a third party (F.A.Z.). To ensure a comprehensive review, we also meticulously examined the reference lists of the reviewed articles, emphasizing the association of RAP with MMM cerebral physiological parameters, neuroimaging, and outcome.

### Data collection

The data field encompasses patient characteristics such as age, population, male/female quantity or percentage, and Glasgow Coma Scale score. Furthermore, it incorporates details about the experimental conditions in the studies, the association of RAP with various MMM parameters, neuroimaging, and patient outcomes. Finally, we incorporated information on individual study limitations and conclusions, particularly pertaining to these RAP-related associations.

### Bias assessment

As the aim of this scoping review was to offer a comprehensive and wide-ranging overview of the existing literature, a formal bias assessment was not carried out.

### Statistical analysis

As the objective of this review was to provide a scoping overview of the existing literature, a meta-analysis was not conducted. This decision was also influenced by the existence of extensive heterogeneity in the study designs and data.

## Results

**FIG. 1.**
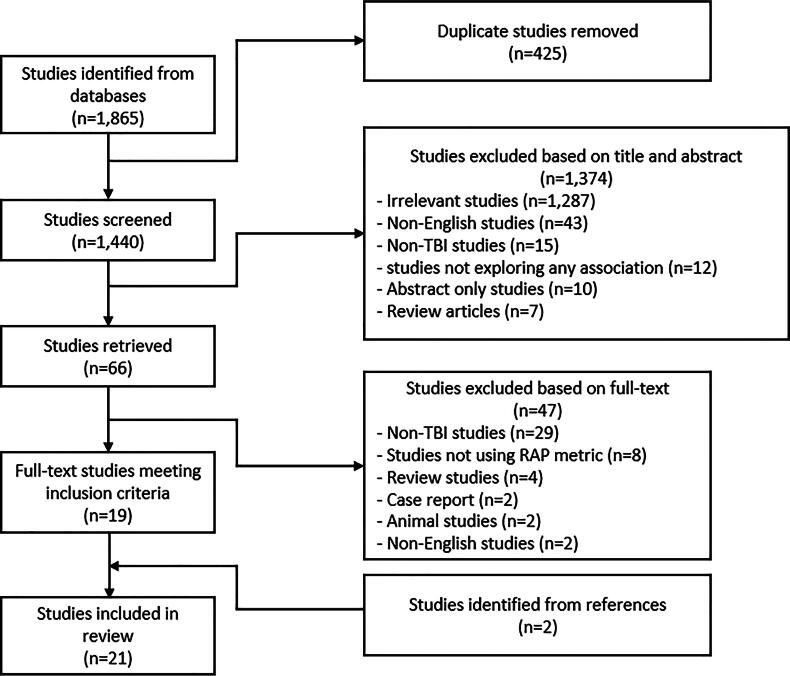
PRISMA Flow Diagram. PRISMA, Preferred Reporting Items for Systematic Reviews and Meta-Analysis.

Using a PRISMA flow diagram, the search and filtration method of this systematic review has been summarized in Figure 1. In total, 1,865 papers were identified from the search strategies applied from the five databases. Among them, 425 studies were identified as duplicates and removed, resulting in 1,440 studies. These 1,440 studies were screened through their title and abstracts. There were 1,374 studies excluded for being irrelevant studies (*n* = 1,287), non-English studies (*n* = 43), non-TBI studies (*n* = 15), studies that are not exploring any association (*n* = 12), conference abstract studies (*n* = 10), or review articles (*n* = 7). Then the 66 retrieved studies’ full-text were thoroughly examined, and 47 studies were excluded based on these criteria—non-TBI studies (*n* = 29), studies that are not using RAP metric (*n* = 8), review studies (*n* = 4), case report (*n* = 2), animal studies (*n* = 2), and non-English studies (*n* = 2). Later, two studies were identified from the reference lists of the included papers, and finally, 21 papers were incorporated into this systematically conducted scoping review. In the sections to follow, we address the studies that evaluate the association between RAP and (1) MMM cerebral physiology, (2) neuroimaging features, and (3) patient outcomes. In addition, they are summarized in Supplementary Tables S1, S2, and S3, respectively.

### Association with continuous MMM cerebral physiological parameters

The change in different continuous MMM cerebral physiological parameters with the change in RAP in TBI patients had been analyzed in 11 studies.^1–3,5–9,15–17^ These observations are based on diverse perspectives and conditions. However, researchers mainly mentioned these values without explicitly demonstrating the direct association of these parameters with RAP but rather signifying the different conditions. In the subsections to follow, we aim to outline the RAP and MMM associations during these condition states.

#### Plateau Waves—RAP and MMM Associations

Four studies explored the values of physiological parameters and RAP during plateau waves of ICP.^5–7^ Sustained and elevated periodic increases in ICP alongside a decrease in cerebral perfusion pressure (CPP) can be observed in TBI patients. These periodic waves are known as plateau waves of ICP, characterized by a gradual elevation to peak pressure, sustained for an extended duration, and then gradually returning to baseline afterward.^6^

Two studies found increased RAP during the plateau wave, which indicated poor CC/CCR.^1,6^ However, instances with higher ICP values during the plateau wave resulted in a lower RAP,^5,7^ from 0.77 ± 0.18 to 0.44 ± 0.19, as mentioned in the study conducted by Czosnyka and colleagues.^7^ This result suggests a state of maximum vasodilation.^5,7^Specifically, in the same study (conducted in 1999), when ICP reached 52.3 mmHg, a reduced positive RAP during the wave was noted,^7^ whereas in cases with even higher ICP levels leading to fatal outcomes, RAP was found to be negative.^5^ Following the occurrence of plateau waves, RAP reverted to its initial state in the majority of observations,^1,7^ except one, where RAP remained the same.^6^

Although comparing the parameters during three stages of the plateau waves (before, during, and after plateau waves), it was observed that impaired RAP was associated with increased ICP and pressure reactivity index (PRx) and a decreased CPP and brain tissue oxygenation (PbtO_2_). They returned close to their previous states after the wave.^1,6,7^ In addition, the index of autoregulation, Mx, and pulsatility index (PI) increased during the plateau wave with a decreased RAP, signifying disturbed autoregulation and increased vascular resistance, respectively.^7^ A decrease in cerebral blood flow velocity (CBFV) was observed during the wave, indicating a diminished ability of cerebral blood vessels to dilate, resulting in higher cerebrovascular resistance. This led to impaired autoregulation.^7^ No significant change in arterial blood pressure (ABP), slow amplitude of slow vasogenic waves of ICP slow (Slow), brain temperature, and pulse amplitude index (PAx) was found.^1,6,7^

Furthermore, in the research conducted by Castellani and colleagues, when comparing patients with and without plateau waves, higher RAP (i.e., poor CC/CCR) associated with slightly higher ICP were observed in the patients with plateau waves. However, PRx, CPP, ABP, and outcome (expressed by the Glasgow outcome scale [GOS]) had no significant change.^6^

#### General ICP Fluctuations—RAP and MMM Associations

The analysis of three studies involved comparing parameters based on the ICP range.^2,8,9^ An increase in ICP was associated with a positive increase in RAP within a specific range of ICP.^2,8,9^ Exceeding that range led to a gradual decrease in RAP.^2,8^ As reported in two studies, RAP exhibited a gradual decrement characteristic when ICP >50 mmHg.^2,8^

In addition, other parameters, including ABP and PRx, progressively increased with both the state of increasing RAP (i.e., higher RAP) and decreasing RAP (i.e., lower RAP). Conversely, CPP, cerebrovascular reactivity (CVR), and PbtO_2_ decreased.^2,8,9^ In one study, an increase in PAx was also observed in the state of low RAP.^9^ Regarding MAP, two studies reported an increment in mean arterial pressure (MAP) in both states of RAP^2,9^; however, one study reported the reduction of MAP with progressive intracranial hypertension when RAP was exhausted, i.e., when RAP was decreasing (i.e., negative changes).^8^

#### Impact of Operative Interventions—RAP and MMM Associations

Comparison of parameters based on different operative approaches is evident in three studies.^15–17^ Two studies observed parameter values before and after decompressive craniectomy.^15,17^ Both analyses reported an improved CCR, i.e., lower RAP with a small positive value following craniectomy. A reduced ICP, MAP, PRx, and Slow were associated with lower RAP. However, CPP and ABP did not exhibit significant changes.^15,17^ The third study focused on the ventriculostomy, which led to a rapid reduction in RAP, signifying improved CCR similar to that achieved with decompressive craniectomy.^16^ Similar to craniectomy, a decrement in ICP and MAP was associated with reduced RAP. Unlike craniectomy, an increase in CPP was associated in the case of ventriculostomy, whereas no significant change in the PRx index was noticed. In addition, PbtO_2_ increased, whereas the change in brain temperature was not significant.^16^

#### Considerations for RAP range in MMM Associations

One study analyzed the values of parameters in two different RAP ranges while observing the respiratory oscillations in CBFV.^3^ When RAP exceeded 0.85, ICP, ABP, and Doppler flow velocity were higher, and CPP was lower compared to cases where RAP was below 0.85. For both RAP ranges, ICP, ABP, Mx, and PI were higher on day 4 compared to day 1 after sedation and mechanical ventilation, whereas CPP was lower on day 4. The increment in Mx and PI was more significant for RAP <0.85.^3^

### Association with neuroimaging features

The association of RAP with neuroimaging was studied in one study, where research was focused on admission CT characteristics.^18^ The analysis was conducted over two distinct time frames—the first 10 days of recording, and the first 48 h of recording. In the context of the first 10 days of recording, certain CT characteristics exhibited significant association with the area under the curve (AUC) for RAP, where higher RAP was observed with an increase in those CT characteristics. These are cortical gyral effacement, lateral ventricle compression, bilateral contusions, cortical subarachnoid hemorrhage (SAH) extent, cortical SAH thickness, and subcortical diffuse axonal injury (DAI).^18^ On the contrary, whereas comparing with RAP, Marshall CT grade, Rotterdam CT grade, Helsinki CT grade, and Stockholm CT grade, these CT grade systems were not significantly associated, whereas midline shift, number of DAI lesions, and total contusion volume were the nonsignificant continuous CT variables.^18^

The AUC for RAP was calculated for each patient by integrating the RAP signal over time using a sequential linear interpolation approach.^18^ In the first 48 h of recording, higher RAP AUC values were associated with the presence of subcortical and corpus callosal DAI lesions. However, none of the continuous CT variables were significantly associated.^18^

### Association with patient outcomes

The relationship between RAP and patient outcome was investigated in nine studies.^4,14,19–25^ In these analyses, observations were conducted regarding the association of RAP with outcome, as well as the association with other parameters in different physiological conditions or outcomes that patients met.

#### Association in the comparison of survival vs. fatal group

Four studies analyzed the association of RAP with survival vs. fatal outcomes.^4,21–23^ In all cases, RAP was significantly lower in the group that met the fatal outcome, which was less than 0.5 for all studies,^21–23^ except one that reported 0.53 for the fatal outcome.^4^ The study conducted by Czosnyka and colleagues reported RAP <0.5 associated with ICP >20 mmHg that differentiated the two groups most significantly in their analysis.^21^

Concerning the relation of RAP with physiological parameters in the context of comparing survival outcomes with the fatal group, lower values of ICP, noninvasive ICP (nICP), and noninvasive amplitude of Slow vasogenic waves of ICPSlow (nSlow) were evident with higher RAP values in the survival group.^4,21^ In contrast, one study observed no significant statistical difference in mean ICP or mean RAP in this comparison. Instead, the dominant ICP pulse type was significantly associated with RAP.^23^ In addition, in moderate cases, RAP >0.5 was associated with relatively higher CPP than the persistent vegetative state (PVS) cases.^21^ On the contrary, this study did not identify a significant difference in ABP between the groups that experienced survival and fatality. The set threshold for the comparison was ABP <70 mmHg. However, it is noteworthy that this specific threshold was effective in distinguishing the group with severe disability from both the survival and fatal groups.^21^

#### Association with other post-TBI Outcomes

Beyond RAP’s association with survival vs. fatal outcome, five studies investigated the association of RAP with outcome from other perspectives.^14,19,20,24,25^ Although comparing post-traumatic hydrocephalus (PTH) and atrophy group, following continuous infusion tests, a notable positive increase in RAP of the PTH group was noticed, which was higher than the atrophy group.^24^ For both of the cases, RAP increased during infusion. However, only the PTH group exhibited depleted CCR.^24^ Another study investigated the potential of RAP in predicting cerebral hemodynamic instability characterized by abnormal rises in ICP following brain injury. It was found that RAP exceeding 0.6 could identify unstable periods with an average positive predictive value (PPV) of 74%.^25^ Asgari and team categorized the patients into “good, ““intermediate, “and “poor” states utilizing the unsupervised training of the Hidden Markov Model (HMM) characterized by ICP, CPP, RAP, and PRx.^19^ A ternary state variable assigned these categories learned through the proposed HMM model training. RAP showed a gradual decrease from a good to a poor state, ending with a mean value of 0.39 in the latter.^19^ In another investigation, based on the values of AMP, RAP, mean ICP, and mean CPP, four states of the TBI patients were defined: state 1, state 2, state 3, and state 4.^20^ RAP was close to zero and did not change with the increase in ICP at state 1, significantly positive at state 2, close to +1 at state 3, and switched to negative at state 4 with a further increase in ICP, marking the terminal stage of the patients.^20^ Zhu and colleagues experimented with the effect of spindle wave in TBI patients, defined as a distinctive pattern observed in electroencephalogram during the nonrapid eye movement sleep stage.^14^ It was found that RAP was significantly lower in the spindle wave group than in the control group.^14^ A similar trend was observed with the GOS extended, with a lower value in the spindle group. In addition, while observing different periods of spindle wave, it was found that RAP during the spindle wave was significantly lower than before and after the spindle wave.^14^

In addition, shedding light on the association of parameters in these observations revealed that a higher value of ICPb (ICP at base) and resistance to CSF outflow (Rout) was associated with an elevated value of RAP in the possible PTH group than in the atrophy group.^24^ However, in terms of detecting unstable cerebral hemodynamic periods, parameters other than RAP did not exhibit significant changes during stable vs. unstable periods.^25^ In the two manuscripts, where different physiological states were described, one had three categories and the other had four.^19,20^ The first study demonstrated a gradual decrement of RAP from good to poor state, which was associated with increased ICP and PRx.^19^ CPP also illustrated a higher value in the intermediate state. However, a lower value was found in the poor state.^19^ In the second analysis with four states, higher RAP was observed in states 1 and 2, followed by lower values in states 3 and 4, even reaching a negative value in state 4.^20^ With this variation in RAP, a rise in ICP and a decrease in CPP were associated across these four states. In state 4, ICP exceeded 65 mmHg and CPP was lower than 30 mmHg.^20^ Finally, in the analysis involving the spindle wave, no significant difference was detected in ICP in the comparison of the spindle wave group to the control group.^14^ However, ICP was lower during the occurrence of the spindle wave compared to the periods before and after the wave.^14^

## Discussion

This systematically conducted scoping review identified some important aspects of the association between RAP and (1) MMM of cerebral physiology, (2) neuroimaging, and (3) patient outcomes. Three states of RAP can be defined to facilitate a more comprehensive discussion on the associations of RAP with the MMM parameters, neuroimaging, and patient outcomes. These states are characterized by the variations in RAP relative to ICP. Supplementary Table S4 presents various RAP values corresponding to different ICP levels, with a primary emphasis on ICP values rather than the patients’ conditions.

In Supplementary Table S4, “Measure 1” indicates the ICP and RAP values where a subsequent measurement shows both higher ICP and RAP (i.e., in “Measure 2”). Conversely, “Measure 2” denotes the ICP and RAP values where a subsequent measurement indicates a higher ICP but a lower RAP (i.e., in “Measure 3”).

It’s evident from the table that in most cases, when ICP surpasses 20 mmHg, RAP begins to decline.^3,4,6,8,15,19^ Before this threshold, RAP generally increases with rising ICP values.^3,4,6,8,15,19^ The studies by Czosnyka et al. in 1999^7^ and Kazimierska et al. in 2021^23^ are exceptions to this trend. Considering this transition, the three states are defined (i.e., state 1, state 2, and state 3). When patients exhibit low and relatively stable RAP levels with minimal changes as ICP increases, this corresponds to state 1. As RAP surpasses 0.5 owing to ICP elevation, the condition is classified as state 2. Finally, when ICP continues to rise beyond 20 mmHg, resulting in lower RAP,^3,4,6,8,15,19^ the condition falls into state 3.

So, the states can be defined as follows:

State 1: RAP is close to zero and exhibits minimal variation with the increase in ICP. State 2: a positive increase in RAP alongside increasing ICP and has a considerable positive value. In this state, RAP exceeds 0.4 and can be seen close to +1. State 3: RAP decreases with the increase in ICP, potentially reaching a negative value when ICP surpasses a critical ICP threshold.

First, in the context of the plateau wave, RAP reached state 2 during the wave.^1,6^ However, if ICP reached a sufficiently high level, state 3 of RAP was observed.^5,7^ In addition, state 2 was associated with elevated ICP and PRx, along with lower values of CPP and CVR during the wave. The positive value of PRx indicated impaired autoregulation.^1,6^ Conversely, in state 3, similar associations were noted for ICP, PRx, CPP, and CVR, as observed in state 2. In addition, disturbed autoregulation and increased vascular resistance were reported as well since increased Mx and PI were observed during the wave.^7^ When comparing patients with and without plateau waves, both groups exhibited elevated RAP, which was positioned in state 2. However, the group experiencing plateau waves had a higher RAP.^7^

Second, in studies focusing on the ICP range, higher RAP was observed with the increase of ICP range, i.e., state 2 of RAP^2,8,9^ and after exceeding a specific value of ICP, RAP kept decreasing, i.e., state 3 of RAP,^2,8^ a pattern reminiscent of findings observed during plateau waves. Regarding other physiological parameters, RAP in state 2 was associated with an increase in MAP, ABP, and PRx and a decrease in CPP, CVR, and PbtO_2_.^2,8,9^ Therefore, these findings, alongside resembling impaired autoregulation as observed in plateau waves, also indicated a reduction in oxygen delivery to brain tissue and impaired CVR in state 2 of RAP. A similar association was observed in state 3 of RAP^2,9^ with an accompanying increase in the PAx.^9^

Third, significant improvement in RAP (i.e., lower RAP with lower ICP) was observed through surgical interventions such as decompressive craniectomy and ventriculostomy, as they were able to shift RAP from state 2 to state 1.^15–17^ During this transition, reductions in ICP, MAP, PRx, and Slow were observed in the case of decompressive craniectomy. Reduced PRx and Slow indicated improved cerebral autoregulation.^15,17^ On the contrary, although ventriculostomy did not yield a significant improvement in PRx, the maintenance of CPP within the normal range suggested an improvement in autoregulation.^18^ In addition, improved oxygen in brain tissue was observed.^18^

Fourth, while comparing survivor and deceased groups of TBI patients, state 3 of RAP was noticed in the latter.^9,14,18^ Higher ICP and nSlow with a lower CPP were associated with the state 3 of RAP in the fatal outcomes, aligning with findings from previous analyses such as plateau waves, ICP range, and operative approaches.^4,21^ Furthermore, in the comparison of the PTH group with the atrophy group, the PTH group showed a RAP value belonging to state 3, associated with higher Rout.^24^ In the case of detecting unstable hemodynamics, state 2 of RAP (greater than 0.6) played an important role,^25^ whereas the presence of spindle waves was found to shift RAP to state 1.^14^ Aside from these studies, two more examined the patients based on their physiological conditions and revealed increased ICP, PRx,^19,20^ and decreased CPP were associated throughout both state 2 and state 3.^19^

Finally, one study analyzed the association of neuroimaging with RAP.^8^ This analysis revealed that toward the conclusion of the initial 10 days of recording, higher values of certain CT characteristics were linked to state 2 of RAP. Furthermore, at the end of the first 48 h of recording, the existence of subcortical and corpus callosal DAI lesions was also associated with the state 2 of RAP.^8^ Overall, throughout the review, the following similarities were found across different conditions, which are differentiated by the states of RAP:

*State 1:* RAP was close to zero. ICP, MAP, PRx, and Slow were the lowest, whereas PbtO_2_ and CPP (balanced) were the highest. These parameter values suggested good CC/CCR, cerebral autoregulation, and ample brain tissue oxygen. This state was observed in TBI patients during the spindle wave.

*State 2:* RAP showed a significant positive value. ICP, MAP, PRx, ABP, and Slow demonstrated higher values than state 1 but lower than state 3. Conversely, CPP, CVR, and PbtO_2_ showed smaller values than state 1 but higher than state 2. These parameter values indicated poor CC/CCR and cerebral autoregulation, inadequate brain tissue oxygen, and impaired CVR. Unstable hemodynamics was detected in this state, and interventions such as decompressive craniectomy and ventriculostomy could shift RAP from state 2 to state 1. Moreover, specific CT characteristics demonstrated elevated values, and the presence of subcortical and corpus callosal DAI lesions was noteworthy in this state.

*State 3:* RAP was more minor than state 2, and with the increase of ICP, RAP reduced, and could reach negative values at one point. ICP, MAP, PRx, ABP, and Slow demonstrated the highest values among the states, whereas CPP, CVR, and PbtO_2_ showed the lowest. Alongside these, Mx, PI, PAx, and Rout were higher than state 2. These parameter values represented exhausted CC/CCR, disturbed cerebral autoregulation, impaired cerebrovascular resistance, insufficient brain tissue oxygen, and compromised CVR. These impairments are more severe than those observed in state 2. Fatal outcomes and PTH outcomes were associated with this state.

The transition from state 2 to state 3, where RAP becomes negative and AMP decreases after ICP exceeds a critical threshold, can be understood physiologically. In State 1, cerebral autoregulation and CVR are intact.^5,20,22^ With the increase of ABP, the cerebral arterial bed constricts to decrease ICP so that cerebral autoregulation remains intact (i.e., negative PRx) and vice versa.^15^ However, in state 2, there will be the presence of impaired cerebral autoregulation and CVR.^15,20^ Physiologically, there is likely increased resistance in cerebral blood vessels, leading to poorer perfusion (i.e., reduced CPP) and brain tissue oxygenation (i.e., reduced PbtO_2_).^6,7,22^ The vessels gradually lose their capability to dilate in response to the change of ABP (i.e., positive PRx).^6,22^ With the further increment of ICP and crossing the critical ICP threshold (i.e., state 3), alongside the decrement of CPP, the cerebral arterioles’ capacity to dilate is exhausted.^5,6,22^ Consequently, they collapse passively.^6,22^ This is associated with the discontinuity of CBF, which leads to further decrement in brain tissue oxygenation.^5^ This is the maximal vasodilation state of the vessels since the CVR is exhausted.^7,21,22^ This signifies a final breakdown in cerebrovascular function, leading to reduced transmission of pulse pressure from the arterial system to the intracranial space.^22^ That’s why even though ICP increases, AMP decreases in this state, and the RAP value becomes negative.

### Limitation and future direction

The limitation section has been broken into two parts—one stating the limitations of the literature explored throughout the review and the other part discussing the limitations of this systematic review.

#### Limitations of the literatures

Some studies experimented with retrospective data, which are often subject to errors and the presence of inconsistency.^5,6,8,9,15,16,19^ In addition, there could be a bias in the data selection since the data might not have been collected with a specific research question. Furthermore, the existence of limitations in controlling for confounding variables could influence the observed associations.^19^ Aside from this, some literature examined small data or excluded too many subjects because of more constraint conditions, which could introduce the lack of generalizability of the findings as the study sample might not be representative of the broader population.^8,9,14–16,18,19,21,25^ The lack of generalizability is also applicable to the case of single-center data, which could also introduce regional biases.^6,14^ Heterogeneity in the data could also be the reason for lack of generalizability.^24^ Furthermore, even though some studies found good associations and results, there is uncertainty regarding their application in the clinical sector.^2–9^

Furthermore, some studies deviated from the common findings, presenting exceptions to the majority.^19,23^ For instance, in the comparison between the survivor and fatal groups, the mean RAP of the fatal group, in most cases, was situated in state 3, accompanied by comparatively higher ICP.^4,5,7,21^ However, Kazimierska and colleagues’ study did not find any significant statistical difference in mean ICP between the two groups; instead, the dominant ICP pulse type was significantly associated with RAP.^23^ The rationale behind this exception was not elucidated in this review. Furthermore, another study noted an initial increase in CPP in state 3 of RAP,^19^ contrary to the majority of studies that reported a decrement in CPP in state 3.^2,4,9,21^ The reason behind the initial CPP increment in this particular study was also not explained.

#### Limitations of this systematic review

This systematic scoping review focuses exclusively on methods involving adult TBI patients and solely incorporates RAP measurements. In addition, non-English studies and animal studies were omitted from the review. Consequently, any potential findings and associations arising from these aspects are not represented in the review.

#### Future direction

The advancement of methods for calculating continuous CC/CCR in TBI necessitates enhanced precision and accuracy for practical implementation in clinical settings. RAP stands out as the extensively researched aspect in this domain, suggesting an opportunity for refinement by delving into its patterns, identifying characteristics, and addressing artifacts to enhance precision. By consolidating all associations of RAP with other continuous MMM cerebral physiological parameters, outcomes, and neuroimaging, this comprehensive review aims to establish a foundation for achieving that improvement. In addition, there is a noticeable gap in the exploration of the neuroimaging domain, with limited studies delving into the correlation between neuroimaging and CC/CCR metrics. Furthermore, certain exceptions identified in specific studies need thorough attention and exploration, particularly if RAP is to be implemented in clinical applications.

## Conclusion

In conclusion, this systematic scoping review offers a comprehensive overview of the correlation between RAP and continuous MMM cerebral physiological parameters, neuroimaging features, and patient outcomes in adult TBI patients. The summary of these associations, categorized into three states of RAP, provides a clear understanding of RAP’s relationship with the mentioned factors. Among these states, the majority of TBI patients appear to be characterized in state 2, and if the situation worsens, they may transition to state 3. State 3 is associated with exhausted CC/CCR, disturbed cerebral autoregulation, impaired cerebrovascular resistance, insufficient brain tissue oxygen, and compromised CVR, representing the most severe conditions among the three states. The insights from this review will serve as a foundation and guide for future research directions, aiming to enhance the precision and clinical implications of utilizing RAP in the care of individuals with TBI so that conditions of the patients from progressing to state 3 from state 2 can be prevented, thereby fatal outcomes can be averted.

## Supplementary Material

Supplementary Table S1

Supplementary Table S2

Supplementary Table S3

Supplementary Table S4

Supplementary Appendix SA1

Supplementary Appendix SA2

## Abbreviations Used

ABP: arterial blood pressure
AMP: pulse amplitude of ICP
AUC: area under the curve
CBFV: cerebral blood flow velocity
CC: cerebral compliance
CCR: continuous compensatory reserve
CPP: cerebral perfusion pressure
CSF: cerebrospinal fluid
CT: computed tomography
CVR: cerebrovascular reactivity
DAI: diffuse axonal injury
GOS: Glasgow Outcome Scale
HMM: hidden Markov model
ICP: intracranial pressure
ICPb: ICP at baseline
MAP: mean arterial pressure
MMM: multi-modal monitoring
MRI: magnetic resonance imaging
Mx: mean flow index
nICP: non-invasive ICP
nSlow: non-invasive Slow
PAx: pulse amplitude index
PbtO2: brain tissue oxygen
PI: pulsatility index
PPV: positive predictive value
PRISMA: Preferred Reporting Items for Systematic Reviews and Meta-Analysis
PRx: pressure reactivity index
PTH: post-traumatic hydrocephalus
RAP: compensatory reserve index (correlation between AMP and ICP)
Rout: resistance to CSF outflow
SAH: subarachnoid hemorrhage
Slow: amplitude of slow vasogenic waves of ICP
TBI: traumatic brain injury
