# Supplementary material for: Association of RAP Compensatory Reserve Index with Continuous Multimodal Monitoring Cerebral Physiology, Neuroimaging, and Patient Outcome in Adult Acute Traumatic Neural Injury: A Scoping Review
Source: Neurotrauma Rep. 2024 Sep 13;5(1):813–23. doi: 10.1089/neur.2024.0058 (PMC11462424; doi:10.1089/neur.2024.0058)
Supplement: Supplementary Table S1 [file neur.2024.0058_Supplemental_Table1.pdf]

**Supplemental Table 1: Association of RAP with continuous MMM cerebral physiologic parameters**

| Article | Patient Population | Experimental Condition | Results | Conclusion | Limitation |
|---|---|---|---|---|---|
| Calviello et al. 2017[5] | 1023 TBI patients. GCS less than 9 (75%) or above 8 (25%). Average patient age was 37 years (range 15–85 years old). Male/female ratio 3:5 | • ICP was monitored prospectively. <br>• A derived variable, wICP, was researched. wICP was calculated as ICP*(1 - RAP). <br>• ICP was monitored with an intraparenchymal sensor. Data were sampled at 100 Hz. <br>• Empirical regression was used to show the nonlinear relationships. <br>• A Kruskal-Wallis statistics number (K) to compare which parameter most strongly differentiated outcome groups | • During plateau waves of ICP and in patients who died from refractory intracranial hypertension, RAP became negative at increased ICP levels. <br>• RAP showed a significant difference between severe disability and favorable outcome groups ($p = 0.004$), which was not the case for ICP and wICP. <br>• RAP switched from positive to negative values at high ICP. | • RAP was the best in patients with good outcome and moderate disability, and was worse in patients with severe disability and non-survivors. <br>• RAP being negative at high ICP hypothetically denoted the final deterioration of cerebral blood flow continuity. | • Data was gathered for over 25 years, encompassing various treatment protocols employed over this period. <br>• The evaluation of the potential miscalculation of the 'wrong ICP' and the 'wrong RAP' using intraparenchymal frontal could not be conducted. |
| Castellani et al. 2009[6] | 444 head injured patients. Male 350. The mean age was 36.3 (±16.4) years. The admission | • Plateau waves were observed in 109 patients among 444 patients. <br>• All the patients were sedated and ventilated. <br>• ICP was monitored using Codman | Comparison between patients without and with plateau waves. <br>• RAP 0.37 [0.18, 0.58], 0.49 [0.26, 0.68] $p \leq 0.020$ <br>• GOS 3 [1, 3] 3 [2, 5] <br>• ABP (mmHg) 95.25 [90.27, 102.00], 96.46 [92.66, 101.98] Ns | Comparison between patients without and with plateau waves. <br>• RAP was worse in the case of patients with plateau waves. <br>• A slightly higher ICP and better CVR (lower RAC) were associated | • Retrospective data. <br>• The patient population and the diagnostic and therapeutic tools had not always been the same. |

| | | | | | |
|---|---|---|---|---|---|
| | median GCS score was 6 (range 3–13). | intraparenchymal microsensors.<br>• Data was analysed using ICM and ICM+. | • ICP (mmHg) 15.00 [11.74, 18.53], 17.57 [14.00, 21.23] <0.000<br>• CPP (mmHg) 79.43 [74.84, 85.91], 79.31 [74.60, 83.30] Ns<br>• PRx 0.046 [-0.041, 0.15], 0.031 [-0.057, 0.12] Ns<br>• RAC -0.12 [-0.29, -0.035], -0.19 [-0.40, -0.073]<br><br>Comparison of the parameters during three stages – before, during and after plateau waves<br>• RAP 0.53 [0.27, 0.78], 0.6 [0.4, 0.8], 0.6 [0.2, 0.8]<br>• ICP (mmHg) 21.0 [16.9, 26.5], 42.2 [36.5, 48.4], 19.4 [15.3, 25.6]<br>• ABP (mmHg) 98.6 [92.0, 108.6], 101.2 [95.0, 109.9], 99.16 [91.6, 107.7]<br>• CPP (mmHg) 77.5 [71.0, 85.2], 59.3 [50.0, 68.8], 78.4 [69, 87.8]<br>• PRx -0.055 [-0.20, 0.13], 0.2 [0.03, 0.54], 0.065 [-0.154, 0.33]<br>• Slow (mmHg) 1.34 [0.6, 2.3], 3.6 [1.9, 6.7], 1.8 [0.6, 6.5]<br>• RAC -0.31 [-0.58, -0.05], -0.32 [-0.46, -0.17], -0.29 [-0.50, -0.089] | with the worse RAP in plateau wave cases.<br>• However, PRx, CPP, ABP and outcome (GOS) had no significant change.<br><br>Comparison of the parameters during three stages – before, during and after plateau waves<br>• RAP worsened (increased) significantly during the wave. After the wave, it remained the same.<br>• Significant increase was found in ICP, PRx, and decrease in CPP during the wave associated with worse RAP. However, unlike RAP, they returned close to their previous states.<br>• An increase in PRx during the wave indicated a loss of CVR (vasodilatation).<br>• No significant change in ABP, Slow and RAC.<br>• No association was found with Marshall Grade (1 to 4). | • CT data was analyzed in about one-quarter of patients only.<br>• Single center TBI data. |

| | | | | | |
|---|---|---|---|---|---|
| Czosnyka et al. 1999[7] | 160 head-injured patients. Mean age 29 years (range 6 to 74 years). Mean GCS score 6 (range 3–13). 120 males. | • Almost 20% of total patients showed plateau waves.<br>• Sixteen episodes of plateau waves in eight patients were recorded and analyzed.<br>• In order to achieve mild hypocapnia, all patients were paralyzed, sedated, and ventilated.<br>• ICM was used to process the data. | Comparison of the parameter values before, during and after the plateau wave –<br>• RAP 0.77 ± 0.18, 0.44 ± 0.19, 0.93 ± 0.05<br>• Mean ICP (mm Hg) 25.9 ±5.7, 52.3 ± 5.6, 21.8 ± 7.2<br>• Mean CPP (mm Hg) 62.5 ± 6.8, 34.1 ± 4.7 65.5 ± 9.4<br>• Mean FV (cm/sec) 52.8 ±19.5, 42.5 ± 22.3, 53.3 ± 15.6<br>• Fva (cm/sec) 44.5 ± 21.5, 55.9 ± 28.1, 32.1 ± 12.3<br>• ICPa (mm Hg) 5.7 ± 2.1, 16.5 ± 4.4, 3.9 ± 1.6<br>• ABPa (mm Hg) 33.7 ± 8.3, 32.0 ± 5.7 31.4 ± 3.5<br>• Mean ABP (mm Hg) 88.5 ± 6.9, 86.5 ± 8.4, 87.3 ± 7.9<br>• CVR (mm Hg/[cm/sec]) 1.34 ± 0.54, 0.98 ± 0.45, 1.72 ± 0.76 0.022<br>• PRx 0.0053 ±0.26, 0.70 ±0.15, 0.052 ± 0.42<br>• Mx 0.067 ± 0.31, 0.497 ± 0.29, 0.218 ± 0.42<br>• PI 1.66 ± 0.31, 2.55 ± 0.49, 1.56 ± 0.35. | • RAP was high before the plateau wave and decreased during the plateau wave, indicating a state of maximum vasodilation and derangement of the normal value.<br>• Unlike RAP, PRx and Mx was preserved during the plateau wave. However, it increased significantly during the wave, indicating a temporary loss of the autoregulatory reserve.<br>• With the exhausted RAP during plateau wave, mean ICP increased, whereas mean CPP and CVR decreased. Mean ABP had no significant change.<br>• The ICP waveform and FV waveform became more pulsatile during plateau waves since ICPa and FVa increased. | |
| Dias et al. 2014[3] | 18 TBI patients, 16 male; mean age 42 years; min 20, max | • Codman (for intraparenchymal ICP), intraparenchymal CBF thermal flow sensor | • During four periods, the parameters were monitored – baseline, during plateau wave, just after plateau | • During the plateau wave, RAP increased but was not significant. After the | |

| | | | | | |
|---|---|---|---|---|---|
| | 66 years. Mean GCS score of 6 (range 3–14) | (Hemedex), Licox (for brain temperature) and near-infrared spectroscopy transcutaneous sensors were used for continuous MMM.<br>• ICM+ was used to collect data.<br>• The primary analysis included the calculation of 10 s averages of ABP, ICP, CPP, $ETCO_2$, CO, $PbtO_2$, CBF, and CVR. Secondary analysis included the calculation of the indices of brain compensatory reserve (RAP) and CVR. | wave, later after plateau wave.<br>• Primary variables<br>ICP (mean ± SD) 17.39 ± 5.3, 47.27 ± 6.47, 16.92 ± 6.85, 15.51 ± 7.46<br><br>ABP (mean ± SD) 107.85 ± 12.93, 108.21 ± 13.97, 109.76 ± 12.90, 108.56 ± 13.01<br><br>CPP (mean ± SD) 90.72 ± 11.37, 61.13 ± 14, 93.12 ± 13.29, 93.18 ± 12.67<br><br>CBF (mean ± SD) 31.64 ± 30.92, 26.18 ± 24.53, 41.15 ± 28.31, 33.67 ± 33.55<br><br>CVR (mean ± SD) 4.88 ± 2.78, 3.97 ± 2.35, 3.64 ± 2.49, 5.23 ± 3.80<br><br>CO (mean ± SD) 53.01 ± 8.45, 50.06 ± 12.78, 50.79 ± 13.11, 51.38 ± 10.51<br><br>$PbtO_2$ (mean ± SD) 21.38 ± 7.95, 16.66 ± 8.67, 20.44 ± 7.34, 20.68 ± 8.01 | plateau wave, RAP decreased.<br>• ICP significantly increased among the primary variables during the plateau wave, while CPP and $PbtO_2$ decreased.<br>• Among the secondary variables, PRx and ORx increased like RAP. Though PRx was statistically significant, ORx wasn't.<br>• Multimodal brain monitoring enables the identification and comprehension of inherent vascular brain phenomena, including plateau waves, and has the potential to assist in the appropriate bedside management of acute head injuries. | |

| | | | | | |
|---|---|---|---|---|---|
| | | | ETCO$_2$ (mean ± SD) 28.12 ± 3.54, 29.93 ± 4.15, 28.89 ± 3.56, 28.26 ± 3.87<br><br>TEMP (mean ± SD) 37.43 ± 0.84, 37.4 ± 0.82, 37.28 ± 0.85, 37.26 ± 0. 90<br>• Secondary variables<br>RAP (mean ± SD) 0.57 ± 0.18, 0.65 ± 0.21, 0.59 ± 0.19, 0.55 ± 0.22<br><br>PRx (mean ± SD) -0.05 ± 0.27, 0.16 ± 0.50, 0.02 ± 0.26, 0.02 ± 0.31<br><br>PAx (mean ± SD) 0.12 ± 0.30, 0.15 ± 0.35, 0.16 ± 0.23, 0.14 ± 0.24<br><br>CBFx (mean ± SD) 0.62 ± 0.26, -0.31 ± 0.39, 0.69 ± 0.24, 0.006 ± 0.19<br><br>COx (mean ± SD) 0.04 ± 0.09, -0.09 ± 0.26, 0.03 ± 0.13, 0.03 ± 0.13<br><br>ORx (mean ± SD) 0.15 ± 0.26, 0.26 ± 0.33, 0.31 ± 0.31, 0.30 ± 0.32. | | |
| Donnelly et al. 2020[2] | 33 sTBI patients with severe | • ICP was monitored with an intraparenchymal | • To investigate the physiologic response due to | • RAP exhibited an increase from low to moderate levels of ICP | • Limited sample size. |

| | | | | | |
|---|---|---|---|---|---|
| | refractory ICH. Mean age 30.3 (sd 12.54). Male 26, GCS less than 8 for 28 patients | sensor. Brain tissue oxygenation was monitored using a Licox probe.<br>• Data were sampled using ICM and ICM+.<br>• Inclusion criteria for patients were as follows: TBI, continuous invasive monitoring of ICP and ABP for a minimum of 12 hours, admission GCS assessment, and availability of 6-month mortality data. | high ICP, three ICP ranges were considered – (0-25), (25-50) and (50-150) mmHg<br>• ICP (mmHg) 17.52 (3.85), 32.80 (2.39), 61.04 (10.79)<br><br>RAP (a.u.) 0.54 (0.20), 0.59 (0.25), 0.46 (0.27)<br><br>CPP (mmHg) 73.90 (9.27), 63.79 (10.41), 39.27 (18.11)<br><br>MAP (mmHg) 91.42 (9.87), 96.61 (9.48), 99.82 (12.55)<br><br>aABP (mmHg) 18.87 (3.32), 19.70 (4.21), 19.85 (5.85)<br><br>PRx (a.u.) (for n = 24) 0.06 (0.26), 0.21 (0.30), 0.57 (0.24)<br><br>$PbtO_2$ (mmHg) (for n = 9) 27.27 (7.32), 20.78 (5.43), 12.68 (7.09) | and subsequently demonstrated a gradual decrease with further elevations in ICP.<br>• Except for $PbtO_2$ and CPP, other parameters (ICP, MAP, aABP, PRx) showed positive associations with RAP in the case of low to moderate levels of ICP. However, they kept increasing/decreasing in the case of further elevations in ICP. | • Increasing the number of patients could have been achieved by loosening the criteria for defining refractory intracranial hypertension.<br>• A lack of comprehensive clinical annotations linked to the monitoring data. |
| Haubrich et al. 2016[1] | 22 head-injured, patients. GCS scores 6.5 ± 3.21 | Patients were analgosedated, and mechanically Ventilated. | Comparison for two cases – RAP <0.85 and RAP ≥ 0.85 (from day 1 to day 4)<br>• ICP 15.72 ± 7.38 to 36.73 ± 11.98 and 12.16 ± 4.01 to 21.25 ± 5.94 | • With RAP being higher (≥ 0.85), ICP, ABP, FV were higher and CPP was lower.<br><br>• For both of the cases of RAP, the | |

| | | | | | |
|---|---|---|---|---|---|
| | | The analysis included transfer function gains of R waves.<br><br>Normal baseline ICP at day 1, subsequently increasing ICP until day 4.<br><br>Intraparenchymal ICP was used for ICP monitoring. | • ABP 91.15 ± 16.08 to 97.50 ± 16.39 and 88.39 ± 12.59 to 95.64 ± 16.41<br>• FVl 55.93 ± 24.39 to 51.89 ± 32.20 and 64.45 ± 23.56 to 72.78 ± 24.07<br>• FVr 55.31 ± 25.56 to 45.88 ± 30.21 and 57.75 ± 20.87 to 63.01 ± 25.40<br>• CPP 81.94 ± 11.19 to 72.48 ± 13.61 and 76.23 ± 12.86 to 75.47 ± 15.41 | parameters – ICP and ABP were higher in day 4 compared to day 1, whereas CPP reduced in day 4.<br>• Mx and PI were also increased in day 4. This increment is more significant for RAP < 0.85.<br>• Patients with RAP <0.85 exhibited significantly higher cerebrovascular pulsatility on day 4 | |
| Timofeev et al. 2008a[14] | 27 TBI patients. Mean age 35 years (range 16–55). Male 22. 20 patients had severe GCS Score <= 8,.<br>7 patients had moderate GCS Score 9–12. | • A specialized neurocritical care unit with ICP and CPP protocol-driven therapy was used to treat all patients, aiming to maintain CPP of >= 60–70 mmHg and ICP of <= 25 mmHg.<br>• When other advanced methods of ICP control or CPP augmentation failed, decompressive craniectomy was performed.<br>• 17 patients have undergone continuous digital ICP recording. | Before and after the decompressive craniectomy –<br>• ICP mm Hg 21.2 [18.7; 24.2], 15.7 [12.3; 19.2]<br>• CPP mm Hg 77.6 [72.8; 80.4], 75.8 [73.2; 82.0]<br>• MAP mm Hg 99.5 [96.2; 102.9], 94.2 [87.9; 98.9]<br>• RAP 0.4 [0.33; 0.68], 0.14 [0.12; 0.22]<br>• PRx 20.03 [20.13; 0.06], 0.14 [0.12; 0.22]<br>• Slow 4.9 [0.7; 7.4], 1.8 [0.4; 6.4] | • After undergoing decompressive craniectomy, the patient had a significant improvement in RAP (i.e. reducing RAP towards zero).<br>• With the reduction of RAP, ICP, MAP, PRx and Slow waves were also reduced.<br>• However, the change in CPP was not significant. | • The proximity of the ICP sensor could affect the postoperative parameter values. As a result, a direct comparison could cause errors.<br>• Retrospective design and small sample size. |

| | | | | | |
|---|---|---|---|---|---|
| | | ICP was monitored using a parenchymal ICP sensor.<br>• SPSS 14.0 software was used to analyze data. | | | |
| Timofeev et al. 2008b[15] | 24 TBI patients. The mean age was 41±16, male 18. GCS was severe for 17 cases, moderate for 4 cases and mild for 3 cases. | • For monitoring ICP, parenchymal ICP sensors were present in the patients.<br>• Ventriculostomy was performed.<br>• Continuous recording of physiological parameters (ICP, CPP, MAP and ICP waveform-derived indices (PRx and RAP) was performed.<br>• The local ICP/CPP driven management protocol influenced the decision to perform ventriculostomy.<br>• Statistical analysis was performed using SPSS 15.0. | Before and after ventriculostomy, for the patients where ICP was sustained after 24hrs (responders) –<br>• RAP 0.20±0.21, 0.07±0.11<br>• ICP (mmHg) 22.7±5.2, 12.5±3.4<br>• CPP (mmHg) 75.5±8.4, 79.6±6.5<br>• MAP (mmHg) 98.1±10.0, 92.1±6<br>• $PbtO_2$ (kPa) 2.3±1.2, 3.2±1.1 | • Ventriculostomy led to a rapid improvement in RAP – reducing its value to zero.<br>• An increase in CPP and $PbtO_2$ was associated with the reduction of RAP. However, ICP and MAP decreased.<br>• No significant change in CVR (the PRx index), and brain temperature. | • Being an observational study, this makes cause–effect interpretations difficult.<br>• Small sample size.<br>• Lack of generalization since patients were selected based on the subjective assessment of ventricular size prior to insertion of the drain.<br>• Representing only a small portion of brain tissue while studying. |
| Whitfield et al. 2001[16] | 26 TBI patients. 21 male. Mean age was | • Patients with head injuries received care following an algorithm for the management of ICP and CPP. | Comparison between pre-operative and post-operative period (sd in parenthesis)–<br>• RAP 0.61 (1.9) 0.25 (0.05)<br>• ICP mean (mmHg) 28 (4) 19 (5) | • Applying decompressive craniectomy significantly improved CCR by reducing RAP to normal. | Cerebral hyperemia was observed only as a contributing factor to ICH. |

| | | | | | |
|---|---|---|---|---|---|
| | 23 years (range 4–59 years). GCS score ranged from 3 to 13, with 18 patients with severe GCS, 5 with moderate, and 3 with initially mild GCS. | • Bifrontal decompressive craniectomy was applied when alternative treatments were unsuccessful in managing post-traumatic ICH.<br>• The applying condition was ICP > 30 mmHg and CPP < 70 mmHg. Or ICP > 35 mmHg.<br>• Around 55% of eligible patients underwent craniectomy. | • ABP (mmHg) 122 (40) 101 (11)<br>• CPP (mmHg) 94 (40) 81 (8)<br>• Slow (mmHg) 1.4 (0.48) 0.44 (0.28) | • Reduction in ICP and Slow were associated with the improved RAP.<br>• ABP and CPP showed no significant change. | |
| Zeiler et al. 2018b[9] | Adult<br>358 TBI patients. 272 males. A median GCS score of 7 (range 3 to 13). The mean age was 40.6 ± 17.2 years (range 16<br>to 89) | • The relationships of this RAC with AMP, ICP, CPP, RAP, PRx and PAx have been explored.<br>• All Signals were recorded using digital data transfer or digitized via A/D converters.<br>• Data was sampled at 50 Hz or higher using ICM+ software.<br>• Statistics were performed utilizing XLSTAT and R statistical software. | ICP < 20 mmHg with progressive arterial hypertension.<br>• RAP starts with close to zero, followed by a subsequent decrease.<br>• With the RAP reduction, MAP, and CPP were also decreased.<br>• As ICP (50 to 60 mm Hg) increased towards the "break-point" in AMP, RAP trended towards +1, signifying the exhaustion of compensatory reserve.<br>• With exhausted compensatory reserve | With low ICP, RAP is reserved. Increase in ICP made RAP worse, and crossing the AMP break point resulted in a negative RAP. | • Retrospective patient populations.<br>• The impact of various vasopressor agents on the cerebrovascular pressure autoregulatory response is not well understood.<br>• The potential RAC monitoring within moderate and severe TBI patients wasnot demonstrated |

| | | | | | |
|---|---|---|---|---|---|
| | | | subsequent decrease in CPP was also seen.<br>• The correlation between RAC and RAP was -0.594. | | clearly in this study. |
| Zeiler et al. 2020[8] | 185 TBI patients. ICP below 15 mmHg, median age 51 years (IQR, 31 to 62.3 years), with a median GCS score of 6 (IQR 3 to 7), and 122 males (77.7%). ICP above 20 mmHg, median age was 54 years (IQR 35.3 to 68.3 years), with a median admission GCS of 7 (IQR 3 to 8), and 19 males (67.9%). | • Subjects included were those with ICP value in the first three days of recording, either below 15 mmHg or above 20 mmHg.<br>• Subjects with EVD-based ICP data were excluded.<br>• ICP was acquired through an intra-parenchymal strain gauge probe and a parenchymal fibre optic pressure sensor. Monitoring of $PbtO_2$ was conducted through invasive parenchymal monitoring.<br>• Data sampling was performed using the ICM+ system. | Group1: mean ICP below 15 mmHg vs group 2: mean ICP above 20 mmHg –<br>• RAP (a.u.) 0.731 [0.560–0.837] 0.710 [0.485–0.783]<br>• ICP (mmHg) 11.0 [8.2–12.9] vs 23.4 [21.9–37.3]<br>• MAP (mmHg) 82.0 [76.2–87.5] vs 89 [80.2–96.8]<br>• CPP (mmHg) 71.8 [66.2–77.6] vs 59.1 [50.0–73.1]<br>• $PbtO_2$ (mmHg)* 27.0 [23.2–33.1] vs 22.1 [18.2–26.2]<br>• PRx (a.u.) − 0.002 [− 0.118 to 0.014] vs 0.206 [− 0.009 to 0.582]<br>• PAx (a.u.) − 0.090 [− 0.208 to 0.095] vs 0.151 [− 0.090 to 0.376] | • With the increase of ICP, RAP was reduced.<br>• With the reduction of RAP, CPP, $PbtO_2$ were also reduced.<br>• On the contrary, MAP, PAx, PRx, increased<br>• Significant disparities were seen in CPP, AMP, CVR, RAP, and $PbtO_2$. | • As this study utilized a multi-center cohort, the influence of different interventions on the recorded cerebral physiology remains unaddressed.<br>• Relatively small data size. As a result, do not provide evidence supporting any directional causal relationship.<br>• No correction for multiple comparisons.<br>• Commenting on the complex interaction among diverse MMM aspects of cerebral physiology was not feasible. |

*aABP, amplitude of arterial blood pressure; ABP, arterial blood pressure; ABPa, fundamental harmonic components for pulse waveforms of ABP; AMP, ICP pulse waveform amplitude; CBF, cerebral blood flow; CBFx, moving correlation between CBF and CPP; CCR, cerebral compensatory reserve; CO, cerebral oximetry; Cox, moving correlation between CO and CPP; CPP, cerebral perfusion pressure; CT, computed tomography; CVR, cerebrovascular reactivity; ETCO$_2$, endtidal CO2; EVD, extraventricular drainage; FV, doppler flow velocity; FVa, fundamental harmonic components for pulse waveforms of FV; FVl, FV left; FVr, FV right; GCS, glasgow coma score; GOS, glasgow outcome scale; GOSE, extended glasgow outcome scale; ICH, intracranial hypertension; ICM+, neuro-intensive care monitoring plus; ICP, intracranial pressure; ICPa, fundamental harmonic components for pulse waveforms of ICP; MAP, mean arterial pressure; MMM, multi-modal monitoring; Mx, the index of autoregulation; Ns, not significant; ORx, oxygen reactivity index; PAx, pulse amplitude index; PbtO$_2$, brain tissue oxygenation; PI, pulsatility index; PRx, pressure reactivity index; RAC, cerebrovascular reactivity; RAP, correlation coefficient between AMP and ICP; SD, standard deviation; Slow, Slow vasogenic waves of ICP; sTBI, severe TBI; TBI, traumatic brain injury; TEMP, brain temperature; wICP, weightedICP;*
