# Supplementary material for: Association of RAP Compensatory Reserve Index with Continuous Multimodal Monitoring Cerebral Physiology, Neuroimaging, and Patient Outcome in Adult Acute Traumatic Neural Injury: A Scoping Review
Source: Neurotrauma Rep. 2024 Sep 13;5(1):813–23. doi: 10.1089/neur.2024.0058 (PMC11462424; doi:10.1089/neur.2024.0058)
Supplement: Supplementary Table S2 [file neur.2024.0058_Supplemental_Table2.pdf]

Supplemental Table 2: Association of RAP with neuroimaging

Article	Patient Population	Experimental Condition	Results	Conclusion	Limitation
Zeiler et al. 2018a[17]	358 adult TBI patients. Mean age was 40.6 ± 17.2 years, 272 male patients. Median admission GCS was 7 (IQR, 3 to 9).	• Only non-craniectomy patients were studied.<br>• RAP was derived using archived high frequency physiologic signals.<br>• Associations of RAP and admission CT injury characteristics were evaluated.<br>• ICP was collected using an intra-parenchymal strain gauge probe.<br>• Each CT scan had a detailed assessment of IC injury. The main focus was mainly on reporting the analysis on the first 10 days of recording.	Association between RAP and admission CT characteristics – first 10 days of recording → • AUC for RAP significantly increased with the increase of the following CT characteristics – cortical gyral effacement, (b) lateral ventricle compression, (c) bilateral contusions, (d) cortical SAH extent, (e) cortical SAH thickness, and (f) subcortical DAI<br>• Among the CT grade systems, the following was not significantly associated – Marshall CT grade, Rotterdam CT grade, Helsinki CT grade, and Stockholm CT grade<br>• The non-significant continuous CT variables were – MLS, Number of DAI lesions, and Total contusion volume	RAP is associated with cerebral CT injury patterns characterized by diffuse injury and edema. A prove that it can potentially be used for compensatory reserve measurement. This serves as evidence supporting its potential use for measuring compensatory reserve.	• A retrospective cohort analysis.<br>• Limited demographics were available

			Association between RAP and admission CT characteristics – first 48 h of recording → • The presence of subcortical and corpus callosal DAI lesions were statistically associated with higher RAP AUC values.<br>• No continuous CT variables were associated with the RAP variables tested.		

AUC, area under the ROC curve; CT, computed tomography; DAI, diffuse axonal injury; GCS, Glasgow coma score; MLS, midline shift; RAP, correlation coefficient between AMP and ICP; SAH, subarachnoid hemorrhage; TBI, traumatic brain injury;
