# Supplementary material for: Association of RAP Compensatory Reserve Index with Continuous Multimodal Monitoring Cerebral Physiology, Neuroimaging, and Patient Outcome in Adult Acute Traumatic Neural Injury: A Scoping Review
Source: Neurotrauma Rep. 2024 Sep 13;5(1):813–23. doi: 10.1089/neur.2024.0058 (PMC11462424; doi:10.1089/neur.2024.0058)
Supplement: Supplementary Table S3 [file neur.2024.0058_Supplemental_Table3.pdf]

Supplemental Table 3: Association of RAP with patient outcomes

Article	Patient Population	Experimental Condition	Results	Conclusion	Limitation
Asgari et al. 2019[18]	379 TBI patients. The average age was 39±17 years. 79% were male. The median admission GCS was 7 with IQR of 4-9.	• The physiological states of the patients were classified into three categories – 'good,' 'intermediate' or 'poor.' This was done by a ternary state variable that was learned through the training.<br>• ICM+ was used to get the hourly values of the parameters.<br>• Exclusion criteria were non-neurological related deaths, vegetative patients due to small numbers, late data monitoring initiation and data discontinuity.<br>• HMM was used to obtain the hourly states of TBI patients	• Parameter values were noted regarding three states – 'good,' 'intermediate,' and 'poor.'<br>• RAP 0.81[0.81-0.82], 0.47[0.46-0.47], 0.39[0.38-0.40]<br>• ICP 14.16[14.08-14.25], 15.88[15.74-16.00], 20.77[20.46-21.08]<br>• CPP 76.98[76.84-77.11], 81.06[80.85-81.28], 69.05[68.73-69.37]<br>• PRx -0.10[(-0.11)-(-0.10)], 0.07[0.06-0.07], 0.41[0.41-0.42]	• RAP showed a gradual decrement from good to poor state, indicating ICP increasing and reaching its critical threshold.<br>• CPP was negatively associated with RAP while comparing good and intermediate states. However, CPP decreased from intermediate to poor state.<br>• PRx had a negative association with RAP.	• Data was retrospective in nature.<br>• 28% of the subjects were excluded following the exclusion criteria.<br>• Absence of other continuous cerebral physiological parameters.<br>• A continuous state variable would be more practical than a discrete state variable.
Budohoski et al. 2012[4]	292 TBI patients. Mean age of 33 ± 16. Mean	• MCA blood FV, ICP and ABP were recorded.	Comparison between the groups who survived vs who died –<br>• RAP (au) 0.63 ± 0.25 vs 0.53 ± 0.28	• The survival group had higher RAP than the group that received a fatal outcome.	Multivariate analysis was not done.

	GCS ± sd was 6 ± 3.	• nICP was calculated using a mathematical model.<br>• AMP, Resp, nSlow, and RAP were calculated (including noninvasive).<br>• ICP was monitored via an intraparenchymal probe. FV was monitored using TCD. ICM+ was used for ICP.	• nRAP (au) 0.56 ± 0.20 vs 0.59 ± 0.17 =0.23<br>• ICP (mmHg) 17.4 ± 8.0 vs 22.9 ± 14<br>• nICP (mmHg) 12.5 ± 5.1 vs 15.3 ± 8.1<br>• Slow (mmHg) 1.35 ± 1.57 vs 1.41 ± 2.32<br>• nSlow (mmHg) 2.09 ± 1.74 vs 2.80 ± 2.37	• No significant change was found in the case of nRAP.<br>• ICP, nICP and nSlow had smaller values (associated with the higher value of RAP) in the survival group.	
Czosnyka et al. 1994[19]	80 head-injured patients.	• One-minute average values of ICP, systemic ABP, CPP, and RAP were recorded.<br>• ICP monitoring was conducted using a saline-filled ventricular or subdural catheter.<br>• Ventilation to procure pCO_2 level between 4.0 and 4.5 kPa was maintained for a minimum of 24 hours and mannitol was used to control ICP below 25 mmHg. If mannitol failed, Propofol was used.<br>• AMP has been evaluated every 8 seconds, using 128	• For CPP below 30 mmHg there is a sharp decrease in amplitude followed by a change RAP from positive to negative values.<br>• The significant decrease in RAP from positive to negative values was observed for increase in ICP from 45 to 70mmHg ($p < 0.01$).<br>• A sharp decrease in RAP from positive to negative was noticed for CPP, decreasing from 35 to 25mmHg ($p < 0.0001$). This indicated a sign of critical disturbance in cerebral circulation.	Considering the values of the parameters, four states were defined.<br>• State 1: RAP did not differ significantly from zero. With the increase of ICP, RAP did not change.<br>• State 2: RAP was significantly positive. Increase in RAP was associated with increase in ICP, ABP and decreasing CPP.<br>• State 3: RAP was close to +1. Similar association like state 2. ICP in this state was 50 to 60 mmHg. CPP was 30 to 50 mmHg.<br>• State 4: RAP switched to negative when ICP	The statistical analysis of the provided clinical materials did not entitle the establishment of fixed ranges for ICP and CPP. Continuous monitoring could be implemented to obtain more precise and detailed information.

		point FFT of the original pressure waveform sampled with a frequency of about 18 Hz.		was higher than 65 mmHg. CPP was lower than 30 mmHg.	
Czosnyka et al. 1996[20]	56 head-injured ventilated patients. 40 males. Mean age 36 years (range 6 to 75 years). Mean GCS Score was 6 (range 3-13).	• The ICP was monitored continuously using a Camino transducer (35 patients) or subdural catheter (21 patients).<br>• The amplitude of ICP pulse waveform was assessed using the fundamental harmonic of the pulse waveform (AMP).<br>• ICM was used for data analysis and recording.<br>• Patients' outcomes were assessed using the GOS at 12 months after the injury.	Percent of time for different conditions with different parameters for moderate/ good, severe disability and PVS/dead group.<br><br>• ICP < 20 mmHg 29%, 55%, 53%.<br>• ABP < 70 mmHg I8%, 7%, 17%.<br>• CPP < 55 mmHg 21%, 30%, 43%.<br>• RAP > 0.5 72%, 62%, 27%.<br>• RAP < 0.5 as a per cent of time when ICP > 20 mmHg 14%, 31%, 75%	• Due to the long period of monitoring, the mean values were averaged, and as a result, they failed to capture significant differences among patients with varying outcomes.<br>• RAP was the most significant associated outcome among the parameters, particularly when considering RAP<0.5 and ICP>20 mmHg.<br>• For the moderate cases, RAP > 0.5 is associated with comparatively higher ICP and CPP than the PVS cases.<br>• In case of RAP < 0.5 was shown mostly in the patients that had ICP > 20 mmHg.	The dataset is small, and a more extensive monitoring of numerous patients is necessary before conducting subgroup analyses based on various pathologies and therapeutic approaches.
Czosnyka et al. 2005[21]	187 TBI patients. Median GCS of 6 (range 3 to 13, 10% of	• Patients were sedated and ventilated to achieve mild hypocapnia.	• RAP: 0.48±0.03 for survivors and 0.33±0.08 for those who died; p < 0.001.	• RAP was independently associated with outcomes. ICP crossing the critical	

	patients with initial GCS > 9). Male 143. Mean age was 36 years; only 7 patients were younger than 15. Age range 6 to 75.	• ICP was monitored continuously using micro transducers.		threshold resulted in low RAP, sometimes even negative.<br>• The critical threshold optimized the differentiation between patients who did not survive and those who did.	
Kazimierska et al. 2021[22]	35 TBI patients. 26 males. Median age of 38 ± 29 years. Median GCS score was 6 ± 4.	• A retrospective single-center data collection process.<br>• The outcome of the patients was using GOS at 3 months after discharge.<br>• ICP was measured using an intraparenchymal sensor.<br>• The signal was processed using ICM+.<br>• The ResNet model was used to classify ICP pulse waveforms into four morphological classes.	Comparison of the parameters between poor outcome and good outcome.<br><br>• ICP [mm Hg] 13.88 ± 5.69 vs 12.31 ± 4.52.<br>• RAP [a.u.] 0.32 ± 0.23 vs 0.46 ± 0.27.<br><br>The association between the dominant ICP pulse type and RAP was<br>• Rp = 0.26, p = 0.004	• No statistically significant differences in mean ICP or mean RAP between good and poor outcome groups.<br>• However, the dominant ICP pulse type was significantly associated with RAP.	Allocating all waveforms from a single patient exclusively to one dataset is done to avoid correlations between datasets that might restrict the model's ability to generalize.
Levrini et al. 2021[23]	50 TBI patients (52 infusion tests). PTH and atrophy had 36 and 16	• A retrospective data where the patient (that had TBI) underwent continuous infusion tests.	Comparison between possible 'PTH' and possible 'atrophy' –<br>• RAPb 0.57 ± 0.18 vs 0.11 ± 0.04<br>• RAPinf 0.9 ± 0.07 vs 0.27 ± 0.11	• Both RAPb and RAPinf were comparatively higher in possible 'PTH.'<br>• High value of ICPb and Rout was also	Patient population was heterogenous, with varying time intervals between investigation and date of TBI

	tests, male 24 and 14, with age 53 ± 17 and 48 ± 16 respectively.	• 52 infusion tests were applied to 50 TBI patients.<br>• Divided into two groups – possible PTH (36 tests) and possible atrophy (14 tests).<br>• R software version 3.5.2. was used for statistical analysis.	• ICPb (mmHg) 9.31 ± 4.12 vs 5.84 ± 3.13<br>• Rout (mmHg/ml/min) 13.41 ± 5.19 vs 4.2 ± 2.03	observed in possible 'PTH.'<br>• In both groups, RAP increased during infusion. However, only possible 'PTH' group had depleted CCR.	
Pineda et al. 2018[24]	Seven head-injured patients. Age > 18 years.	• ICP data was randomly chosen and segmented into 1 hour. Hours were then categorized as either stable, which contained no sharp rises in ICP, or unstable, which contained ≥1 sharp rise.<br>• The Mann–Whitney U and the paired t-test was applied to find differences between stable and unstable RAP data	• Unstable cerebral hemodynamic periods were identified by RAP > 0.6, with an average PPV of 74%.<br>• ICP exceeded 20 mmHg in 41.3% and 45.2%, respectively, for the case of stable and unstable periods.	RAP served as a reliable indicator of ICP dynamics, remained unaffected by sensor drift, and could more effectively distinguish periods of instability compared to ICP or AMP alone.	A small set of patient sample.
Zhu et al. 2022[25]	60 TBI<br>Age between 26 and 84 years old with a mean of (53.52 ± 15.25). 41 males. The	• Evaluating TBI patients with spindle waves (from EEG) - 20 cases had spindle waves, 40 didn't have (control group).<br>• Patients with known pregnancy or the presence of	While comparing the baseline information of the two groups, the following were found – Spindle wave group vs control group.<br>• ICP 20.48 ± 7.54 vs 19.88 ± 7.73 0.77<br>• RAP 0.21 ± 0.14 vs 0.33 ± 0.15 0.004	• RAP was significantly lower in the spindle wave group than in the control group. A similar pattern was observed in the case of GOSE. However, ICP was not associated with RAP since no	• Small sample size from single center.<br>• Occurrence of the spindle wave remains unknow.

	admission GCS scores were between 3 and 14 with a mean of 8.02 ± 3.31 points	contraindications to ICP monitoring such were excluded.	• 6-month GOSE 4.90 ± 2.10 vs 3.68 ± 2.04 0.04<br><br>Three stages were considered for comparison – before the appearance of the spindle wave, during the appearance of the spindle wave, and after the disappearance of the spindle wave.<br>• ICP 23.31 ± 7.80, 18.11 ± 7.67, 19.86 ± 8.46<br>• RAP 0.31 ± 0.13, 0.04 ± 0.18, 0.18 ± 0.12	difference was found for ICP.<br>• RAP during the spindle wave was also significantly lower than that before and after the spindle wave, the same scenario for ICP.	

ABP, arterial blood pressure; AMP, ICP pulse waveform amplitude; CCR, cerebral compensatory reserve; CPP, cerebral perfusion pressure; EEG, electroencephalogram; FFT, fast Fourier transform; FV, doppler flow velocity; GCS, Glasgow coma score; GOS, Glasgow outcome scale; GOSE, extended Glasgow outcome scale; HMM, Hidden Markov Model; ICM+, neuro-intensive care monitoring plus; ICP, intracranial pressure; ICPb, ICP at baseline; MCA, middle cerebral arteries; nSlow, non-invasive Slow; PRx, pressure reactivity index; PTH, post-traumatic hydrocephalus; PVS, persistent vegetative state; RAP, correlation coefficient between AMP and ICP; RAPb, RAP at baseline; RAPinf, RAP at infusion; Resp, amplitude of the respiratory component; Rout, resistance to CSF outflow; sd, standard deviation; TBI, traumatic brain injury; TCD, transcranial Doppler;
