# Supplementary material for: Association of RAP Compensatory Reserve Index with Continuous Multimodal Monitoring Cerebral Physiology, Neuroimaging, and Patient Outcome in Adult Acute Traumatic Neural Injury: A Scoping Review
Source: Neurotrauma Rep. 2024 Sep 13;5(1):813–23. doi: 10.1089/neur.2024.0058 (PMC11462424; doi:10.1089/neur.2024.0058)
Supplement: Supplementary Table S4 [file neur.2024.0058_Supplemental_Table4.pdf]

Supplemental Table 4: Association between ICP and RAP values.

	ICP (mmHg)			RAP		
**Studies**	**Measure 1**	**Measure 2**	**Measure 3**	**Measure 1**	**Measure 2**	**Measure 3**
Asgari et al 2019[18]	14.16	15.88	20.77	0.81	0.47	0.39
Budohoski et al. 2012[4]		17.4 ± 8.0	22.9 ± 14		0.63 ± 0.25	0.53 ± 0.28
Castellani et al. 2009[6]	15.00	17.57		0.37	0.49	
Czosnyka et al. 1999[7]	25.9 ± 5.7	52.3 ± 5.6	21.8 ± 7.2	0.77 ± 0.18	0.44 ± 0.19	0.93 ± 0.05
Haubrich et al. 2016[1]	12.16 ± 4.01	21.25 ± 5.94		RAP < 0.85	RAP ≥ 0.85	
Kazimierska et al. 2021[22]		12.31 ± 4.52	13.88 ± 5.69		0.46 ± 0.27	0.32 ± 0.23
Timofeev et al. 2008a[14]	12.5±3.4	22.7±5.2		0.07±0.11	0.20±0.21	
Zeiler et al. 2020[9]		11.0	23.4		0.731	0.710

ICP, intracranial pressure; RAP, correlation coefficient between AMP and ICP;
