# Supplementary material for: Association of RAP Compensatory Reserve Index with Continuous Multimodal Monitoring Cerebral Physiology, Neuroimaging, and Patient Outcome in Adult Acute Traumatic Neural Injury: A Scoping Review
Source: Neurotrauma Rep. 2024 Sep 13;5(1):813–23. doi: 10.1089/neur.2024.0058 (PMC11462424; doi:10.1089/neur.2024.0058)
Supplement: Supplementary Appendix SA2 [file neur.2024.0058_Supplemental_AppendixA2.pdf]

Appendix B

Following are the search strings that were used while searching in database.

Search Strategy for PubMed/Medline, Embase, Scopus, and Cochrane Library

"Traumatic Brain Injury" OR TBI OR "Brain Trauma" OR "Traumatic Encephalopathy" OR "Traumatic Encephalopathies" OR "Traumatic Brain Injuries" OR TBIs OR "Head injury" OR "Cranial trauma" OR "Traumatic Intracranial Injury" OR "Intracranial Trauma" OR "Closed head trauma" OR "Head trauma" OR "Traumatic Head injury" OR "Brain concussion" OR "Cerebral concussion" OR "Cephalohematoma" OR "Brain contusion" OR "Fractured skull" OR "Skull fracture" OR "Neural injury" OR "Diffuse Axonal Injury"

AND

RAP OR "Compensatory Reserve" OR "Pearson’s Correlation" OR "Correlation between ICP pulse amplitude and ICP" OR "Pearson’s Correlation between ICP pulse amplitude and cerebral perfusion pressure" OR "Compensatory Reserve Index" OR "RAP-weighted mean ICP" OR "Correlation between AMP and ICP" OR "Compensatory Reserve metric"

AND

"Intracranial Pressure" OR ICP OR "Partial pressure of brain tissue oxygenation" OR PbtO2OR "Cerebral Temperature" OR "Regional Cerebral Blood Flow" OR "Cerebral Blood Flow" OR rCBF OR CBF OR "jugular bulb venous oximetry" OR SvjO2 OR "Cerebral Microdialysis" OR CMD OR "Arterio-Jugular Oxygen Content" OR AJDO2 OR "Arterio–venous difference" OR "arteriovenous difference in oxygen" OR AVDO2 OR "Autoregulation index" OR ARI OR "Cerebral Blood Flow Velocity" OR CBFv OR "Cerebral blood volume" OR CBV OR "Cerebral circulation" OR "Cerebral Flow metabolism" OR "Cerebral hemodynamics" OR "Cerebral homeostasis" OR "Cerebral metabolic rate for oxygen" OR CMRO2 OR "Cerebral metabolism" OR "Cerebral microcirculation" OR "Cerebral Oximetry index" OR Cox OR "Cerebral perfusion" OR "Cerebral Perfusion Pressure" OR CPP OR "Cerebral pressure" OR "Cerebral pressure autoregulation" OR "Cerebral spinal reserve capacity" OR "Cerebral Oximetry index" OR "Cerebral vascular reactivity" OR "Cerebral vascular resistance" OR "Cerebral vasculature" OR "Cerebral vasoconstriction" OR "Cerebral vasodilation" OR "Cerebral vasomotor responsiveness" OR "Cerebral Vasoreactivity" OR "Cerebral Vessel Diameter" OR "Cerebrovascular control" OR "Cerebrovascular Function" OR "Cerebrovascular reactivity" OR "Cortical blood flow" OR "Cortical laser doppler" OR "Cortical perfusion" OR "dense array electroencephalography" OR dEEG OR "Diastolic flow index" OR Dx OR "Diffuse Correlation Spectroscopy" OR DCS OR "Diffusion Weighted Imaging index" OR DWI OR "Dynamic autoregulatory index" OR Dax OR Electrocorticography OR EcOG OR Electroencephalography OR EEG OR "Flow velocity" OR FV OR "hemoglobin volume index" OR HBx OR "Jugular bulb saturation" OR SjO2 OR "Jugular venous oxygen saturation" OR "Arteriovenous oxygen difference" OR a-vO2 OR SvjO2 OR SvO2 OR "Kety–Schmidt technique" OR "Laser Doppler flowmetry" OR LDF OR "Mean flow index" OR Mx OR "Mean transit time" OR MTT OR "Near infrared spectrometry" OR NIRS OR "Neurovascular autoregulation" OR "Neurovascular coupling" OR "Neurovascular reactivity" OR "orthostatic hypotension test" OR OHT OR "oxygen extraction fraction" OR OEF OR "Perfusion-weighted imaging index" OR "Pressure Reactivity index" OR PRx OR "Projection pursuit regression" OR PPR OR "Pulsatile Reactivity index" OR Pax OR "Pulsatility Index" OR "regional cerebral oxygen saturation" OR rSO2 OR "Spatially resolved NIRS" OR "stereo electroencephalography" OR sEEG OR "Systolic flow index" OR Sx OR "Thermal Diffusion" OR TDx OR "Time to peak" OR TTP OR "Thigh cuff deflation technique" OR TCDT OR "transient hyperemic response test" OR THRT OR "tissue hemoglobin index" OR tHbx OR "Transcranial Doppler" OR TCD OR "Transcranial doppler index"

OR TCDx OR "Tissue Oxygenation index" OR Tox OR "Transcranial Doppler Sonography" OR "Transfer function analysis" OR TF OR TFA OR "focal tissue oxygen tension" OR "computed tomography" OR CT OR "Enhanced computed tomography" OR "Enhanced CT" OR "Magnetic Resonance Imaging" OR MRI OR "Functional Imaging" OR fMRI OR "Susceptibility Weighted Imaging" OR SWI OR "Gradient Echo" OR GRE OR "Fluid Attenuated Inversion Recovery" OR FLAIR OR "Functional MRI" OR "Positron Emission Tomography" OR PET OR "Single Photon Emission Computed Tomography" OR SPECT OR "Functional near-infrared spectroscopy" OR fNIRS OR "Magnetoencephalography" OR MEG OR "Magnetization Transfer Imaging" OR MTI OR Elastography OR "Glasgow Outcome Scale" OR "Trauma Severity Indices" OR "Glasgow Outcome Scale Extended" OR GOS OR GOSE OR "Patient Outcome Assessments" OR "Outcome Assessments" OR "Functional outcome scores" OR "Injury Severity Score" OR "Probability of Death Score" OR "Maximum AIS" OR "Disability Rating Scale" OR DRS OR "Functional Independence Measure" OR FIM OR "Community Integration Questionnaire" OR CIQ OR "Functional Status Examination" OR FSE OR "Neuro behavioural Function Inventory" OR "Rey Complex Figure" OR "Controlled Oral Word Association Test" OR COWAT OR "Symbol Digit Modalities" OR "Grooved Pegboard" OR "Quality of Life" OR QOL OR "Quality of Life after Brain Injury" OR "Marshal CT" OR "Stockholm CT" OR "Rotterdam CT" OR "Abbreviated Injury Scale" OR AIS OR QOLIBRI OR GOSE-E OR NOS-TBI OR "Neurological Outcome Scale for Traumatic Brain Injury"

Search Strategy for Biosis

ALL=("Traumatic Brain Injury") OR ALL=(TBI) OR ALL=("Brain Trauma") OR ALL=("Traumatic Encephalopathy") OR ALL=("Traumatic Encephalopathies") OR ALL=("Traumatic Brain Injuries") OR ALL=(TBIs) OR ALL=("Head injury") OR ALL=("Cranial trauma") OR ALL=("Traumatic Intracranial Injury") OR ALL=("Intracranial Trauma") OR ALL=("Closed head trauma") OR ALL=("Head trauma") OR ALL=("Traumatic Head injury") OR ALL=("Brain concussion") OR ALL=("Cerebral concussion") OR ALL=("Cephalohematoma") OR ALL=("Brain contusion") OR ALL=("Fractured skull") OR ALL=("Skull fracture") OR ALL=("Neural injury") OR ALL=("Diffuse Axonal Injury")

AND

ALL=(RAP) OR ALL=("Compensatory Reserve") OR ALL=("Pearson’s Correlation") OR ALL=("Correlation between ICP pulse amplitude and ICP") OR ALL=("Pearson’s Correlation between ICP pulse amplitude and cerebral perfusion pressure") OR ALL=("Compensatory Reserve Index") OR ALL=("RAP-weighted mean ICP") OR ALL=("Correlation between AMP and ICP") OR ALL=("Compensatory Reserve metric")

AND

ALL=("Intracranial Pressure") OR ALL=(ICP) OR ALL=("Partial pressure of brain tissue oxygenation") OR ALL=(PbtO2) OR ALL=("Cerebral Temperature") OR ALL=("Regional Cerebral Blood Flow") OR ALL=("Cerebral Blood Flow") OR ALL=(rCBF) OR ALL=(CBF) OR ALL=("jugular bulb venous oximetry") OR ALL=(SvjO2) OR ALL=("Cerebral Microdialysis") OR ALL=(CMD) OR ALL=("Arterio-Jugular Oxygen Content") OR ALL=(AJDO2) OR ALL=("Arterio–venous difference") OR ALL=("arteriovenous difference in oxygen") OR ALL=(AVDO2) OR ALL=("Autoregulation index") OR ALL=(ARI) OR ALL=("Cerebral Blood Flow Velocity") OR ALL=(CBFv) OR ALL=("Cerebral blood volume") OR ALL=(CBV) OR ALL=("Cerebral circulation") OR ALL=("Cerebral Flow metabolism") OR ALL=("Cerebral hemodynamics") OR ALL=("Cerebral homeostasis") OR ALL=("Cerebral metabolic rate for oxygen") OR ALL=(CMRO2) OR ALL=("Cerebral metabolism") OR ALL=("Cerebral microcirculation") OR ALL=("Cerebral Oximetry index") OR ALL=(Cox) OR ALL=("Cerebral perfusion") OR ALL=("Cerebral Perfusion Pressure") OR ALL=(CPP) OR ALL=("Cerebral pressure") OR ALL=("Cerebral pressure autoregulation") OR ALL=("Cerebral spinal reserve capacity") OR ALL=("Cerebral Oximetry index") OR ALL=("Cerebral vascular reactivity") OR ALL=("Cerebral vascular

resistance") OR ALL=("Cerebral vasculature") OR ALL=("Cerebral vasoconstriction") OR ALL=("Cerebral vasodilation") OR ALL=("Cerebral vasomotor responsiveness") OR ALL=("Cerebral Vasoreactivity") OR ALL=("Cerebral Vessel Diameter") OR ALL=("Cerebrovascular control") OR ALL=("Cerebrovascular Function") OR ALL=("Cerebrovascular reactivity") OR ALL=("Cortical blood flow") OR ALL=("Cortical laser doppler") OR ALL=("Cortical perfusion") OR ALL=("dense array electroencephalography") OR ALL=(dEEG) OR ALL=("Diastolic flow index") OR ALL=(Dx) OR ALL=("Diffuse Correlation Spectroscopy") OR ALL=(DCS) OR ALL=("Diffusion Weighted Imaging index") OR ALL=(DWI) OR ALL=("Dynamic autoregulatory index") OR ALL=(Dax) OR ALL=(Electrocorticography) OR ALL=(EcOG) OR ALL=(Electroencephalography) OR ALL=(EEG) OR ALL=("Flow velocity") OR ALL=(FV) OR ALL=("hemoglobin volume index") OR ALL=(HBx) OR ALL=("Jugular bulb saturation") OR ALL=(SjO2) OR ALL=("Jugular venous oxygen saturation") OR ALL=("Arteriovenous oxygen difference") OR ALL=(a-vO2) OR ALL=(SvjO2) OR ALL=(SvO2) OR ALL=("Kety–Schmidt technique") OR ALL=("Laser Doppler flowmetry") OR ALL=(LDF) OR ALL=("Mean flow index") OR ALL=(Mx) OR ALL=("Mean transit time") OR ALL=(MTT) OR ALL=("Near infrared spectrometry") OR ALL=(NIRS) OR ALL=("Neurovascular autoregulation") OR ALL=("Neurovascular coupling") OR ALL=("Neurovascular reactivity") OR ALL=("orthostatic hypotension test") OR ALL=(OHT) OR ALL=("oxygen extraction fraction") OR ALL=(OEF) OR ALL=("Perfusion-weighted imaging index") OR ALL=("Pressure Reactivity index") OR ALL=(PRx) OR ALL=("Projection pursuit regression") OR ALL=(PPR) OR ALL=("Pulsatile Reactivity index") OR ALL=(Pax) OR ALL=("Pulsatility Index") OR ALL=("regional cerebral oxygen saturation") OR ALL=(rSO2) OR ALL=("Spatially resolved NIRS") OR ALL=("stereo electroencephalography") OR ALL=(sEEG) OR ALL=("Systolic flow index") OR ALL=(Sx) OR ALL=("Thermal Diffusion") OR ALL=(TDx) OR ALL=("Time to peak") OR ALL=(TTP) OR ALL=("Thigh cuff deflation technique") OR ALL=(TCDT) OR ALL=("transient hyperemic response test") OR ALL=(THRT) OR ALL=("tissue hemoglobin index") OR ALL=(tHbx) OR ALL=("Transcranial Doppler") OR ALL=(TCD) OR ALL=("Transcranial doppler index") OR ALL=(TCDx) OR ALL=("Tissue Oxygenation index") OR ALL=(Tox) OR ALL=("Transcranial Doppler Sonography") OR ALL=("Transfer function analysis") OR ALL=(TF) OR ALL=(TFA) OR ALL=("focal tissue oxygen tension") OR ALL=("computed tomography") OR ALL=(CT) OR ALL=("Enhanced computed tomography") OR ALL=("Enhanced CT") OR ALL=("Magnetic Resonance Imaging") OR ALL=(MRI) OR ALL=("Functional Imaging") OR ALL=(fMRI) OR ALL=("Susceptibility Weighted Imaging") OR ALL=(SWI) OR ALL=("Gradient Echo") OR ALL=(GRE) OR ALL=("Fluid Attenuated Inversion Recovery") OR ALL=(FLAIR) OR ALL=("Functional MRI") OR ALL=("Positron Emission Tomography") OR ALL=(PET) OR ALL=("Single Photon Emission Computed Tomography") OR ALL=(SPECT) OR ALL=("Functional near-infrared spectroscopy") OR ALL=(fNIRS) OR ALL=("Magnetoencephalography") OR ALL=(MEG) OR ALL=("Magnetization Transfer Imaging") OR ALL=(MTI) OR ALL=(Elastography) OR ALL=("Glasgow Outcome Scale") OR ALL=("Trauma Severity Indices") OR ALL=("Glasgow Outcome Scale Extended") OR ALL=(GOS) OR ALL=(GOSE) OR ALL=("Patient Outcome Assessments") OR ALL=("Outcome Assessments") OR ALL=("Functional outcome scores") OR ALL=("Injury Severity Score") OR ALL=("Probability of Death Score") OR ALL=("Maximum AIS") OR ALL=("Disability Rating Scale") OR ALL=(DRS) OR ALL=("Functional Independence Measure") OR ALL=(FIM) OR ALL=("Community Integration Questionnaire") OR ALL=(CIQ) OR ALL=("Functional Status Examination") OR ALL=(FSE) OR ALL=("Neuro behavioural Function Inventory") OR ALL=("Rey Complex Figure") OR ALL=("Controlled Oral Word Association Test") OR ALL=(COWAT) OR ALL=("Symbol Digit Modalities") OR ALL=("Grooved Pegboard") OR ALL=("Quality of Life") OR ALL=(QOL) OR ALL=("Quality of Life after Brain Injury") OR ALL=("Marshal CT") OR ALL=("Stockholm CT") OR ALL=("Rotterdam CT") OR ALL=("Abbreviated Injury Scale") OR ALL=(AIS) OR ALL=(QOLIBRI) OR ALL=(GOSE-E) OR ALL=(NOS-TBI) OR ALL=("Neurological Outcome Scale for Traumatic Brain Injury")
